# Correction: Wang et al. Parametric Formula for Stress Concentration Factor of Fillet Weld Joints with Spline Bead Profile. *Materials* 2020, *13*, 4639

**DOI:** 10.3390/ma14092433

**Published:** 2021-05-07

**Authors:** Yixun Wang, Yuxiao Luo, Seiichiro Tsutsumi

**Affiliations:** 1 Joining and Welding Research Institute, Osaka University, Osaka 567-0047, Japan; wang.yixun@jwri.osaka-u.ac.jp; 2 Department of Structural Engineering, Tongji University, Shanghai 200092, China; 1410210@tongji.edu.cn

The authors wish to revise the following from pages 16–18 in the text of Appendix B [1] due to the presence of incorrect information.

The correct text is shown below:

## Appendix B

Formulas for calculating SCFs for T-shape welded joint and cruciform welded joint under tensile and bending stress.

(1) T-shape welded joint under tensile stress:
  (A5) $$K_{t}=1+1.39418\cdot f\left(r_{1}/t\right)\cdot f\left(\theta_{1}\right)\cdot f\left(\left(r_{1}/t\right)\cdot \left(\theta_{1}\right)\right)$$
  where:
  $$\begin{array}{ll} f\left(r_{1}/t\right)= & 1.824174\cdot {\left(r_{1}/t\right)}^{-0.145045}+6.400210\cdot {\left(r_{1}/t\right)}^{3}-6.802011\cdot {\left(r_{1}/t\right)}^{2}+3.277059\cdot \left(r_{1}/t\right)\;\\ & -2.692331 \end{array}$$
  $$f\left(\theta_{1}\right)=-2.778232\times 10^{-1}\cdot {\left(\pi -\theta_{1}\right)}^{3}+1.531999\cdot {\left(\pi -\theta_{1}\right)}^{2}-3.394907\cdot \left(\pi -\theta_{1}\right)+4.967130$$
  $$\begin{array}{ll} f\left(\left(r_{1}/t\right)\cdot \left(\theta_{1}\right)\right)= & -1.001612\times 10^{1}\cdot {\left(\left(r_{1}/t\right)\cdot \left(\pi -\theta_{1}\right)\right)}^{3}+1.279269\times 10^{1}\cdot {\left(\left(r_{1}/t\right)\cdot \left(\pi -\theta_{1}\right)\right)}^{2}\\ & +5.761117\cdot \left(\left(r_{1}/t\right)\cdot \left(\pi -\theta_{1}\right)\right)\;+1.181649 \end{array}$$
(2) T-shape welded joint under bending stress:
  (A6) $$\begin{array}{ll} K_{t}= & 1-1.129553\cdot f\left(T/t\right)\cdot f\left(r_{1}/t\right)\cdot f\left(\theta_{1}\right)\cdot f\left(\left(r_{1}/t\right)\cdot \left(\theta_{1}\right)\right)\cdot f\left(L_{1}/t\right)\cdot f\left(L_{2}/t\right)\\ & \cdot \;\;\;f\left(\left(L_{1}/t\right)\cdot \left(L_{2}/t\right)\right)\cdot f\left(H/t\right) \end{array}$$
  where:
  $$f\left(T/t\right)=-9.622916\times 10^{-3}\cdot {\left(T/t\right)}^{3}-3.157009\times 10^{-2}\cdot {\left(T/t\right)}^{2}+1.848086\times 10^{-1}\cdot \left(T/t\right)+2.433139$$
  $$\begin{array}{ll} f\left(r_{1}/t\right)= & 6.477191\times 10^{-1}\cdot {\left(r_{1}/t\right)}^{-0.258620}+2.860640\cdot {\left(r_{1}/t\right)}^{3}-3.689862\cdot {\left(r_{1}/t\right)}^{2}\\ & +2.089786\cdot \left(r_{1}/t\right)-1.238719 \end{array}$$
  $$\begin{array}{ll} f\left(\theta_{1}\right)= & 3.450624\times 10^{-3}\cdot {\left(\pi -\theta_{1}\right)}^{3}-3.531382\times 10^{-2}\cdot {\left(\pi -\theta_{1}\right)}^{2}+6.008736\times 10^{-2}\cdot \left(\pi -\theta_{1}\right)\\ & +8.576512\times 10^{-2} \end{array}$$
  $$\begin{array}{ll} f\left(\left(r_{1}/t\right)\cdot \left(\theta_{1}\right)\right)= & 3.532021\cdot {\left(\left(r_{1}/t\right)\cdot \left(\pi -\theta_{1}\right)\right)}^{5.127981}-8.442514\cdot {\left(\left(r_{1}/t\right)\cdot \left(\pi -\theta_{1}\right)\right)}^{3}\\ & +\;8.645182\cdot {\left(\left(r_{1}/t\right)\cdot \left(\pi -\theta_{1}\right)\right)}^{2}+\;\;1.481668\cdot \left(\left(r_{1}/t\right)\cdot \left(\pi -\theta_{1}\right)\right)+3.814145\times 10^{-1} \end{array}$$
  $$\begin{array}{ll} f\left(L_{1}/t\right)= & -1.140609\times 10^{1}\cdot {\left(L_{1}/t\right)}^{3.036113}+7.436459\times 10^{-2}\cdot {\left(L_{1}/t\right)}^{4}+1.188223\times 10^{1}\cdot {\left(L_{1}/t\right)}^{3}\\ & -8.150334\times 10^{-1}\cdot {\left(L_{1}/t\right)}^{2}\;+2.933742\times 10^{-1}\cdot \left(L_{1}/t\right)+2.718308\times 10^{-2} \end{array}$$
  $$\begin{array}{ll} f\left(L_{2}/t\right)= & 1.919634\times 10^{1}\cdot {\left(L_{2}/t\right)}^{-0.1508265}-7.315526\times 10^{-1}\cdot {\left(L_{2}/t\right)}^{4}+4.967357\cdot {\left(L_{2}/t\right)}^{3}\\ & -8.382442\cdot {\left(L_{2}/t\right)}^{2}-5.482638\cdot \left(L_{2}/t\right)+4.359819\times 10^{1} \end{array}$$
  $$\begin{array}{ll} f\left(\left(L_{1}/t\right)\cdot \left(L_{2}/t\right)\right)= & -1.823407\times 10^{-1}\cdot {\left(\left(L_{1}/t\right)\cdot \left(L_{2}/t\right)\right)}^{3}+1.469586\cdot {\left(\left(L_{1}/t\right)\cdot \left(L_{2}/t\right)\right)}^{2}\\ & -\;5.862377\cdot \left(\left(L_{1}/t\right)\cdot \left(L_{2}/t\right)\right)-8.301064 \end{array}$$
  $$f\left(H/t\right)=-1.913752\times 10^{1}\cdot {\left(H/t\right)}^{3}+7.690410\cdot {\left(H/t\right)}^{2}-5.994878\times 10^{-1}\cdot \left(H/t\right)+1.201826$$
(3) Cruciform welded joint under tensile stress:
  (A7) $$\begin{array}{ll} K_{t}= & 1+9.355385\times 10^{-1}\cdot f\left(T/t\right)\cdot f\left(r_{1}/t\right)\cdot f\left(\theta_{1}\right)\cdot f\left(\left(r_{1}/t\right)\cdot \left(\theta_{1}\right)\right)\cdot f\left(L_{1}/t\right)\cdot f\left(L_{2}/t\right)\cdot \\ & f\left(\left(L_{1}/t\right)\cdot \left(L_{2}/t\right)\right)\cdot f\left(H/t\right) \end{array}$$
  where:
  $$f\left(T/t\right)=-4.241098\times 10^{-1}\cdot {\left(T/t\right)}^{3}+1.392836\cdot {\left(T/t\right)}^{2}-1.072866\cdot \left(T/t\right)+5.223873$$
  $$\begin{array}{ll} f\left(r_{1}/t\right)= & -2.214616\cdot {\left(r_{1}/t\right)}^{0.5081972}+3.365455\cdot {\left(r_{1}/t\right)}^{3}-4.439046\cdot {\left(r_{1}/t\right)}^{2}+3.726006\cdot \left(r_{1}/t\right)\\ & +4.033244\times 10^{-1} \end{array}$$
  $$f\left(\theta_{1}\right)=-7.886656\times 10^{-1}\cdot {\left(\pi -\theta_{1}\right)}^{3}+2.966534\cdot {\left(\pi -\theta_{1}\right)}^{2}-5.878541\cdot \left(\pi -\theta_{1}\right)+1.772818\times 10^{1}$$
  $$\begin{array}{ll} f\left(\left(r_{1}/t\right)\cdot \left(\theta_{1}\right)\right)= & 1.557408\cdot {\left(\left(r_{1}/t\right)\cdot \left(\pi -\theta_{1}\right)\right)}^{-0.1158765}+3.821716{\left(\left(r_{1}/t\right)\cdot \left(\pi -\theta_{1}\right)\right)}^{3}\\ & -5.354226\cdot {\left(\left(r_{1}/t\right)\cdot \left(\pi -\theta_{1}\right)\right)}^{2}\;\;\;+7.153383\cdot \left(\left(r_{1}/t\right)\cdot \left(\pi -\theta_{1}\right)\right)-1.529427 \end{array}$$
  $$\begin{array}{ll} f\left(L_{1}/t\right)= & 8.158610\cdot {\left(L_{1}/t\right)}^{1.944849}-1.90075\times 10^{-2}\cdot {\left(L_{1}/t\right)}^{4}+2.021235\times 10^{-1}\cdot {\left(L_{1}/t\right)}^{3}-7.977936\cdot {\left(L_{1}/t\right)}^{2}\\ & -4.569099\times 10^{-1}\cdot \left(L_{1}/t\right)+1.108427\times 10^{-1} \end{array}$$
  $$\begin{array}{ll} f\left(L_{2}/t\right)= & 1.020926\times 10^{1}\cdot {\left(L_{2}/t\right)}^{0.09585178}+3.856400\times 10^{-1}\cdot {\left(L_{2}/t\right)}^{4}-2.432551\cdot {\left(L_{2}/t\right)}^{3}\\ & +6.069561\cdot {\left(L_{2}/t\right)}^{2}-8.045796\cdot \left(L_{2}/t\right)-5.364472 \end{array}$$
  $$\begin{array}{ll} f\left(\left(L_{1}/t\right)\cdot \left(L_{2}/t\right)\right)= & -2.421137\times 10^{-1}\cdot {\left(\left(L_{1}/t\right)\cdot \left(L_{2}/t\right)\right)}^{3}+\;5.706142\times 10^{-1}\cdot {\left(\left(L_{1}/t\right)\cdot \left(L_{2}/t\right)\right)}^{2}\\ & -2.159502\times 10^{1}\cdot \left(\left(L_{1}/t\right)\cdot \left(L_{2}/t\right)\right)\;-2.240262 \end{array}$$
  $$f\left(H/t\right)=4.996101\cdot {\left(H/t\right)}^{3}-1.461031\cdot {\left(H/t\right)}^{2}-1.874919\times 10^{-1}\cdot \left(H/t\right)-1.083093$$
(4) Cruciform welded joint under bending stress:
  (A8) $$K_{t}=1+1.20077\cdot f\left(r_{1}/t\right)\cdot f\left(\theta_{1}\right)\cdot f\left(\left(r_{1}/t\right)\cdot \left(\theta_{1}\right)\right)\cdot f\left(L_{1}/t\right)\cdot f\left(L_{2}/t\right)\cdot f\left(\left(L_{1}/t\right)\cdot \left(L_{2}/t\right)\right)$$
  where:
  $$f\left(r_{1}/t\right)=1.245919\cdot {\left(r_{1}/t\right)}^{-0.1788861}+5.187295\cdot {\left(r_{1}/t\right)}^{3}-5.888376\cdot {\left(r_{1}/t\right)}^{2}+2.948041\cdot \left(r_{1}/t\right)-2.024161$$
  $$f\left(\theta_{1}\right)=4.518553\times 10^{-1}\cdot {\left(\pi -\theta_{1}\right)}^{3}-4.169720\cdot {\left(\pi -\theta_{1}\right)}^{2}+6.471859\cdot \left(\pi -\theta_{1}\right)+1.232356\times 10^{1}$$
  $$\begin{array}{ll} f\left(\left(r_{1}/t\right)\cdot \left(\theta_{1}\right)\right)= & 6.195167\cdot {\left(\left(r_{1}/t\right)\cdot \left(\pi -\theta_{1}\right)\right)}^{1.958767}+2.102936\times 10^{-1}\cdot {\left(\left(r_{1}/t\right)\cdot \left(\pi -\theta_{1}\right)\right)}^{3}\\ & -6.258362\cdot {\left(\left(r_{1}/t\right)\cdot \left(\pi -\theta_{1}\right)\right)}^{2}\;+\;4.307112\times 10^{-2}\cdot \left(\left(r_{1}/t\right)\cdot \left(\pi -\theta_{1}\right)\right)+1.578839\times 10^{-2} \end{array}$$
  $$f\left(L_{1}/t\right)=1.069048\times 10^{-1}\cdot {\left(L_{1}/t\right)}^{3}+4.896665\times 10^{-1}\cdot {\left(L_{1}/t\right)}^{2}-3.181390\cdot \left(L_{1}/t\right)+1.187674\times 10^{1}$$
  $$\begin{array}{ll} f\left(L_{2}/t\right)= & 6.217977\cdot {\left(L_{2}/t\right)}^{2.938345}+8.220848\times 10^{-2}\cdot {\left(L_{2}/t\right)}^{4}-5.872895\cdot {\left(L_{2}/t\right)}^{3}-5.835022\times 10^{-1}\cdot {\left(L_{2}/t\right)}^{2}\\ & +1.809699\times 10^{-1}\cdot \left(L_{2}/t\right)+2.220297\times 10^{-2} \end{array}$$
  $$\begin{array}{ll} f\left(\left(L_{1}/t\right)\cdot \left(L_{2}/t\right)\right)= & -9.639892\times 10^{-2}\cdot {\left(\left(L_{1}/t\right)\cdot \left(L_{2}/t\right)\right)}^{3}-2.213564\times 10^{-1}\cdot {\left(\left(L_{1}/t\right)\cdot \left(L_{2}/t\right)\right)}^{2}\\ & +5.660853\cdot \left(\left(L_{1}/t\right)\cdot \left(L_{2}/t\right)\right)\;+3.300823\times 10^{1} \end{array}$$

The previously given text is shown below:

## Appendix B

Formulas for calculating SCFs for T-shape welded joint and cruciform welded joint under tensile and bending stress.

(1) T-shape welded joint under tensile stress:
  (A5) $$K_{t}=1+1.394\cdot f\left(r_{1}/t\right)\cdot f\left(\theta_{1}\right)\cdot f\left(\left(r_{1}/t\right)\cdot \left(\theta_{1}\right)\right)$$
  where:
  $$f\left(r_{1}/t\right)=1.824\cdot {\left(r_{1}/t\right)}^{-0.145}+6.400\cdot {\left(r_{1}/t\right)}^{3}-6.802\cdot {\left(r_{1}/t\right)}^{2}+3.277\cdot \left(r_{1}/t\right)-2.692$$
  $$f\left(\theta_{1}\right)=-0.278\cdot {\left(\pi -\theta_{1}\right)}^{3}+1.532\cdot {\left(\pi -\theta_{1}\right)}^{2}-3.395\cdot \left(\pi -\theta_{1}\right)+4.967$$
  $$\begin{array}{ll} f\left(\left(r_{1}/t\right)\cdot \left(\theta_{1}\right)\right)= & -10.016\cdot {\left(\left(r_{1}/t\right)\cdot \left(\pi -\theta_{1}\right)\right)}^{3}+12.793\cdot {\left(\left(r_{1}/t\right)\cdot \left(\pi -\theta_{1}\right)\right)}^{2}+\\ & 5.761\cdot \left(\left(r_{1}/t\right)\cdot \left(\pi -\theta_{1}\right)\right)\;+1.182 \end{array}$$
(2) T-shape welded joint under bending stress:
  (A6) $$\begin{array}{ll} K_{t}= & 1-1.130\cdot f\left(T/t\right)\cdot f\left(r_{1}/t\right)\cdot f\left(\theta_{1}\right)\cdot f\left(\left(r_{1}/t\right)\cdot \left(\theta_{1}\right)\right)\cdot f\left(L_{1}/t\right)\cdot f\left(L_{2}/t\right)\cdot \\ & f\left(\left(L_{1}/t\right)\cdot \left(L_{2}/t\right)\right)\cdot f\left(H/t\right) \end{array}$$
  where:
  $$f\left(T/t\right)=-0.010\cdot {\left(T/t\right)}^{3}-0.032\cdot {\left(T/t\right)}^{2}+0.185\cdot \left(T/t\right)+2.433$$
  $$f\left(r_{1}/t\right)=0.648\cdot {\left(r_{1}/t\right)}^{-0.259}+2.861\cdot {\left(r_{1}/t\right)}^{3}-3.690\cdot {\left(r_{1}/t\right)}^{2}+2.090\cdot \left(r_{1}/t\right)-1.239$$
  $$f\left(\theta_{1}\right)=0.003\cdot {\left(\pi -\theta_{1}\right)}^{3}-0.035\cdot {\left(\pi -\theta_{1}\right)}^{2}+0.060\cdot \left(\pi -\theta_{1}\right)+0.086$$
  $$\begin{array}{ll} f\left(\left(r_{1}/t\right)\cdot \left(\theta_{1}\right)\right)= & 3.532\cdot {\left(\left(r_{1}/t\right)\cdot \left(\pi -\theta_{1}\right)\right)}^{5.128}-8.443\cdot {\left(\left(r_{1}/t\right)\cdot \left(\pi -\theta_{1}\right)\right)}^{3}+\;\;\\ & 8.645\cdot {\left(\left(r_{1}/t\right)\cdot \left(\pi -\theta_{1}\right)\right)}^{2}+\;\;1.482\cdot \left(\left(r_{1}/t\right)\cdot \left(\pi -\theta_{1}\right)\right)+0.381 \end{array}$$
  $$f\left(L_{1}/t\right)=-11.406\cdot {\left(L_{1}/t\right)}^{3.036}+0.074\cdot {\left(L_{1}/t\right)}^{4}+11.882\cdot {\left(L_{1}/t\right)}^{3}-0.815\cdot {\left(L_{1}/t\right)}^{2}+0.293\cdot \left(L_{1}/t\right)+0.027$$
  $$f\left(L_{2}/t\right)=19.196\cdot {\left(L_{2}/t\right)}^{-0.151}-0.732\cdot {\left(L_{2}/t\right)}^{4}+4.967\cdot {\left(L_{2}/t\right)}^{3}-8.382\cdot {\left(L_{2}/t\right)}^{2}-5.483\cdot \left(L_{2}/t\right)+43.598$$
  $$f\left(\left(L_{1}/t\right)\cdot \left(L_{2}/t\right)\right)=-0.182\cdot {\left(\left(L_{1}/t\right)\cdot \left(L_{2}/t\right)\right)}^{3}+1.470\cdot {\left(\left(L_{1}/t\right)\cdot \left(L_{2}/t\right)\right)}^{2}-\;5.862\cdot \left(\left(L_{1}/t\right)\cdot \left(L_{2}/t\right)\right)-8.301$$
  $$f\left(H/t\right)=-19.138\cdot {\left(H/t\right)}^{3}+7.690\cdot {\left(H/t\right)}^{2}-0.599\cdot \left(H/t\right)+1.202$$
(3) Cruciform welded joint under tensile stress:
  (A7) $$\begin{array}{ll} K_{t}= & 1+0.936\cdot f\left(T/t\right)\cdot f\left(r_{1}/t\right)\cdot f\left(\theta_{1}\right)\cdot f\left(\left(r_{1}/t\right)\cdot \left(\theta_{1}\right)\right)\cdot f\left(L_{1}/t\right)\cdot f\left(L_{2}/t\right)\cdot \\ & f\left(\left(L_{1}/t\right)\cdot \left(L_{2}/t\right)\right)\cdot f\left(H/t\right) \end{array}$$
  where:
  $$f\left(T/t\right)=-0.424\cdot {\left(T/t\right)}^{3}+1.393\cdot {\left(T/t\right)}^{2}-1.073\cdot \left(T/t\right)+5.224$$
  $$f\left(r_{1}/t\right)=-2.215\cdot {\left(r_{1}/t\right)}^{0.508}+3.365\cdot {\left(r_{1}/t\right)}^{3}-4.439\cdot {\left(r_{1}/t\right)}^{2}+3.726\cdot \left(r_{1}/t\right)+0.403$$
  $$f\left(\theta_{1}\right)=-0.789\cdot {\left(\pi -\theta_{1}\right)}^{3}+2.967\cdot {\left(\pi -\theta_{1}\right)}^{2}-5.879\cdot \left(\pi -\theta_{1}\right)+17.728$$
  $$\begin{array}{ll} f\left(\left(r_{1}/t\right)\cdot \left(\theta_{1}\right)\right)= & 1.557\cdot {\left(\left(r_{1}/t\right)\cdot \left(\pi -\theta_{1}\right)\right)}^{-0.116}+3.822\cdot {\left(\left(r_{1}/t\right)\cdot \left(\pi -\theta_{1}\right)\right)}^{3}-5.354\cdot {\left(\left(r_{1}/t\right)\cdot \left(\pi -\theta_{1}\right)\right)}^{2}+\\ & 7.153\cdot \left(\left(r_{1}/t\right)\cdot \left(\pi -\theta_{1}\right)\right)-1.529 \end{array}$$
  $$f\left(L_{1}/t\right)=8.159\cdot {\left(L_{1}/t\right)}^{1.945}-0.019\cdot {\left(L_{1}/t\right)}^{4}+0.202\cdot {\left(L_{1}/t\right)}^{3}-7.978\cdot {\left(L_{1}/t\right)}^{2}-\;0.457\cdot \left(L_{1}/t\right)+0.111$$
  $$f\left(L_{2}/t\right)=10.209\cdot {\left(L_{2}/t\right)}^{0.096}+0.386\cdot {\left(L_{2}/t\right)}^{4}-2.433\cdot {\left(L_{2}/t\right)}^{3}+6.070\cdot {\left(L_{2}/t\right)}^{2}-8.046\cdot \left(L_{2}/t\right)-5.364$$
  $$f\left(H/t\right)=4.996\cdot {\left(H/t\right)}^{3}-1.461\cdot {\left(H/t\right)}^{2}-0.187\cdot \left(H/t\right)-1.083$$
  $$f\left(\left(L_{1}/t\right)\cdot \left(L_{2}/t\right)\right)=-0.242\cdot {\left(\left(L_{1}/t\right)\cdot \left(L_{2}/t\right)\right)}^{3}+\;0.571\cdot {\left(\left(L_{1}/t\right)\cdot \left(L_{2}/t\right)\right)}^{2}-21.595\cdot \left(\left(L_{1}/t\right)\cdot \left(L_{2}/t\right)\right)-2.240$$
(4) Cruciform welded joint under bending stress:
  (A8) $$\begin{array}{ll} K_{t}= & 1+1.201\cdot f\left(r_{1}/t\right)\cdot f\left(\theta_{1}\right)\cdot f\left(\left(r_{1}/t\right)\cdot \left(\theta_{1}\right)\right)\cdot f\left(L_{1}/t\right)\cdot f\left(L_{2}/t\right)\cdot \\ & f\left(\left(L_{1}/t\right)\cdot \left(L_{2}/t\right)\right) \end{array}$$
  where:
  $$f\left(r_{1}/t\right)=1.246\cdot {\left(r_{1}/t\right)}^{-0.179}+5.187\cdot {\left(r_{1}/t\right)}^{3}-5.888\cdot {\left(r_{1}/t\right)}^{2}+2.948\cdot \left(r_{1}/t\right)-2.024$$
  $$f\left(\theta_{1}\right)=0.452\cdot {\left(\pi -\theta_{1}\right)}^{3}-4.170\cdot {\left(\pi -\theta_{1}\right)}^{2}+6.472\cdot \left(\pi -\theta_{1}\right)+12.324$$
  $$\begin{array}{ll} f\left(\left(r_{1}/t\right)\cdot \left(\theta_{1}\right)\right)= & 6.195\cdot {\left(\left(r_{1}/t\right)\cdot \left(\pi -\theta_{1}\right)\right)}^{1.959}+0.210\cdot {\left(\left(r_{1}/t\right)\cdot \left(\pi -\theta_{1}\right)\right)}^{3}-\;\;6.258\cdot {\left(\left(r_{1}/t\right)\cdot \left(\pi -\theta_{1}\right)\right)}^{2}+\\ & 0.043\cdot \left(\left(r_{1}/t\right)\cdot \left(\pi -\theta_{1}\right)\right)+0.016 \end{array}$$
  $$f\left(L_{1}/t\right)=0.107\cdot {\left(L_{1}/t\right)}^{3}+0.490\cdot {\left(L_{1}/t\right)}^{2}-3.181\cdot \left(L_{1}/t\right)+11.877$$
  $$f\left(L_{2}/t\right)=6.218\cdot {\left(L_{2}/t\right)}^{2.938}+0.082\cdot {\left(L_{2}/t\right)}^{4}-5.873\cdot {\left(L_{2}/t\right)}^{3}-0.584\cdot {\left(L_{2}/t\right)}^{2}+0.181\cdot \left(L_{2}/t\right)+0.022$$
  $$f\left(\left(L_{1}/t\right)\cdot \left(L_{2}/t\right)\right)=-0.096\cdot {\left(\left(L_{1}/t\right)\cdot \left(L_{2}/t\right)\right)}^{3}-0.221\cdot {\left(\left(L_{1}/t\right)\cdot \left(L_{2}/t\right)\right)}^{2}+5.661\cdot \left(\left(L_{1}/t\right)\cdot \left(L_{2}/t\right)\right)+33.008$$

The authors would like to apologize for any inconvenience caused to the readers by these changes.
